# Estimation of (co)variance components for very large datasets and complex single-step genomic models

**DOI:** 10.1186/s12711-025-01006-9

**Published:** 2025-10-30

**Authors:** Matias Bermann, Andres Legarra, Ignacio Aguilar, Alejandra Alvarez-Munera, Ignacy Misztal, Daniela Lourenco

**Affiliations:** 1 Department of Animal and Dairy Science, University of Georgia, Athens, GA 30602 USA; 2 Council On Dairy Cattle Breeding, Bowie, MD 20716 USA; 3 Instituto Nacional de Investigación Agropecuaria (INIA), 11500 Montevideo, Uruguay

## Abstract

**Background:**

Variance components of linear mixed models should be estimated with all the data and information available for a specific statistical model to avoid bias. Due to computational limitations, the estimation for large datasets or complex models is often carried out by subsetting the data, removing genomic information, or simplifying the statistical model. Monte Carlo REML (MC-REML) is a method developed to lift computational limitations, but so far there was no extension for genomic information under the single-step genomic methods. In this study, we extended MC-REML to include large genomic information.

**Results:**

We developed a method to estimate variance components named Monte Carlo single-step genomic REML (MC-ss-GREML). The core of the method includes repeatedly simulating breeding values under a ssGBLUP model and solving the mixed model equations to approximate traces involving prediction error variances. The REML optimization strategies include Expectation Maximization and Average Information. We tested the accuracy of MC-ss-GREML with a three-trait growth model in beef cattle with maternal effects, with 14 parameters to estimate. The data set had 100,000 animals in the pedigree, of which about 33,000 had records, and 10,000 were genotyped. There were no differences in estimates between MC-ss-GREML and ss-GREML with the exact calculation of the traces (exact ss-GREML). MC-ss-GREML took 14% of the computing time and used 1% of the memory compared to the exact ss-GREML. We tested the computing performance of MC-ss-GREML by estimating variance components in a birth weight model, with much larger data that included 7 million animals in the pedigree, from which 330,000 were genotyped. The estimation converged in 11 rounds and took 121 h, with a peak memory usage of 53 Gb.

**Conclusions:**

The new method, MC-ss-GREML, can estimate variance components with large datasets including many genotyped individuals, at affordable time and memory costs.

## Background

Variance components should be estimated with all the data and information available for a specific statistical model to avoid a biased estimation [1]. However, for large datasets or complex models, the estimation is carried out using a data subset or simplifying the model by removing genomic information or traits due to computational limitations. The consequences of this practice are incorrect variance components, which can lead to a biased estimated genetic merit and accuracy, a wrong estimate of the genetic progress, and an incorrect calculation of selection indexes. The latter could lead to wrong selection decisions if (co)variances between traits change over time.

The most popular computational methods for variance components estimation (VCE) are the maximization of the restricted likelihood (henceforth referred to as REML) and Markov Chain Monte Carlo (MCMC) methods, especially the Gibbs sampler [2]. For large datasets and complex models, REML suffers from time limitation due to the need to invert the mixed-model equations coefficient matrix $\left(\mathbf{C}\right)$, whereas MCMC methods require a large number of samples to obtain reasonable estimates [2]. When genomic information is used via single-step genomic BLUP (ssGBLUP) Mixed Model Equations (MME), REML becomes prohibitive due to memory requirements. A Gibbs sampler for a model equivalent to ssGBLUP is theoretically possible to implement for very large datasets [3]. However, to our knowledge, no experiences with large datasets with millions of animals in the pedigree and hundreds of thousands of genotypes have been reported. On the other hand, Monte Carlo REML (MC-REML) was developed to overcome timing and memory limitations in REML [4–7]. Again, no studies reported an implementation of MC-REML with genomic information. MC-REML involves simulating breeding values, records, and solving MME to obtain gradients needed for Expectation Maximization (EM) and Average Information REML. This is straightforward for pedigree-based models, for purely genomic (all individuals genotyped) models, but not for ssGBLUP models. Pimentel et al. [8] developed a method to simulate breeding values for ssGBLUP models to check how pedigree errors affect genetic evaluations. In this study, we aimed to combine the MC-REML with an improved version of the method of Pimentel et al. [8] to develop an algorithm for VCE for large and complex ssGBLUP models. We called this method Monte Carlo single-step GREML (MC-ss-GREML).

### Methods

## Theory

We assume a general linear model:

1 $${\mathbf{y}} = {\mathbf{Xb}} + {\mathbf{Wp}} + {\mathbf{Zu}} + {\mathbf{e}},$$

where $\mathbf{y}$ is the vector of phenotypes, $\mathbf{b}$ is the vector of fixed effects, $\mathbf{p}$ is the vector of non-genetic random effects distributed as $\mathbf{p}\sim N\left(0,{\mathbf{P}}_{0}\otimes \mathbf{I}\right)$, $\mathbf{u}$ is the vector of genetic random effects distributed as $\mathbf{u}\sim N\left(0,{\mathbf{G}}_{0}\otimes \mathbf{H}\right)$, $\mathbf{e}$ is the vector of errors distributed as $\mathbf{e}\sim N\left(0,{\mathbf{R}}_{0}\otimes \mathbf{I}\right)$, and $\mathbf{X}$, $\mathbf{W}$, and $\mathbf{Z}$ are incidence matrices. $\mathbf{H}$ is defined as in Legarra et al. [9], and its inverse is presented in Aguilar et al. [10] and Christensen and Lund [11]. Letting $\mathbf{V}=Var\left(\mathbf{y}\right)=\mathbf{W}\left({\mathbf{P}}_{0}\otimes \mathbf{I}\right){\mathbf{W^{\prime}}}+\mathbf{Z}\left({\mathbf{G}}_{0}\otimes \mathbf{H}\right){\mathbf{Z^{\prime}}}+{\mathbf{R}}_{0}\otimes \mathbf{I}$ and assuming $\mathbf{X}$ to be a full-rank matrix, the restricted log-likelihood from [12] can be written as:

2 $$- 2 \ell \left( {{\varvec{\uptheta}}} \right) = \log \left| {\mathbf{V}} \right| + \log \left| {{\mathbf{X^{\prime}V}}^{ - 1} {\mathbf{X}}} \right| + {\mathbf{y^{\prime}Py}},$$

where $\mathbf{P}={\mathbf{V}}^{-1}-{\mathbf{V}}^{-1}\mathbf{X}{\left({\mathbf{X^{\prime}}}{\mathbf{V}}^{-1}\mathbf{X}\right)}^{-1}{\mathbf{X^{\prime}}}{\mathbf{V}}^{-1}$, and ${\varvec{\uptheta}}$ is the matrix of all (co)variance components in the model. The first partial derivatives of $-2{\ell}\left({\varvec{\uptheta}}\right)$ with respect to the *ij*th element of ${\varvec{\uptheta}}$ are:

3 $$- 2\frac{{\partial \ell \left( {{\varvec{\uptheta}}} \right)}}{{\partial {\uptheta }_{ij} }} = {\text{tr}}\left( {\frac{{\partial {\mathbf{V}}}}{{\partial {\uptheta }_{ij} }}{\mathbf{P}}} \right) - {\mathbf{y^{\prime}P}}\frac{{\partial {\mathbf{V}}}}{{\partial {\uptheta }_{ij} }}{\mathbf{Py}}.$$

Assume that vector $\mathbf{k}$ represents the *k*th group of correlated random effects of the model, except for the error. Then, $Var\left(\mathbf{k}\right)={{\varvec{\uptheta}}}_{k}\otimes \mathbf{K}$, and Eq. (3) results in [13]:

4 $$- 2\frac{{\partial \ell \left( {{\varvec{\uptheta}}} \right)}}{{\partial {{\varvec{\uptheta}}}_{k} }} = q_{k} {{\varvec{\uptheta}}}_{k}^{ - 1} - {{\varvec{\uptheta}}}_{k}^{ - 1} \left( {{\mathbf{T}}_{k} + {\mathbf{S}}_{k} } \right){{\varvec{\uptheta}}}_{k}^{ - 1} ,$$

where ${q}_{k}$ is the number of levels of $\mathbf{k}$, and

5 $${\mathbf{T}}_{{k_{ij} }} = {\text{tr}}\left( {{\mathbf{K}}^{ - 1} {\mathbf{C}}^{{k_{i} k_{j} }} } \right),$$

where $\mathbf{C}$ denotes the coefficient matrix of the mixed-model equations (MME) and ${\mathbf{C}}^{{k}_{i}{k}_{j}}$ is the block of ${\mathbf{C}}^{-1}$ pertaining to the *k*th effect and the *ij*th trait combination. Also,

6 $${\mathbf{S}}_{{k_{ij} }} = {\hat{\mathbf{k}}}_{i}^{\prime} {\mathbf{K}}^{ - 1} {\hat{\mathbf{k}}}_{j} ,$$

where ${\widehat{\mathbf{k}}}_{i}$ is the estimate of $\mathbf{k}$ for the *i*th trait.

The first partial derivatives with respect to the residual variance components are:

7 $$- 2\frac{{\partial \ell \left( {{\varvec{\uptheta}}} \right)}}{{\partial {\mathbf{R}}_{{0_{ij} }} }} = {\text{tr}}\left( {{\mathbf{R}}_{ij} {\mathbf{R}}_{l}^{ - 1} } \right) - {\text{tr}}\left( {{\mathbf{R}}_{l}^{ - 1} {\mathbf{R}}_{ij} {\mathbf{R}}_{l}^{ - 1} {\mathbf{QC}}^{ - 1} {\mathbf{Q^{\prime}}}} \right) - {\hat{\mathbf{e}}}^{\prime} {\mathbf{R}}_{l}^{ - 1} {\mathbf{R}}_{ij} {\mathbf{R}}_{l}^{ - 1} {\hat{\mathbf{e}}},$$

where ${\mathbf{R}}_{ij}$ is an indicator matrix containing 1 in the *ij*th position and 0 elsewhere, ${\mathbf{R}}_{l}$ is the estimate of ${\mathbf{R}}_{0}$ in the *l*th iteration, and $$\mathbf{Q}=\left[\begin{array}{ccc}\mathbf{X}& \mathbf{W}& \mathbf{Z}\end{array}\right]$$.

The second partial derivates of $-2{\ell}\left({\varvec{\uptheta}}\right)$ with respect to the *ij*th and *kl*th elements of ${\varvec{\uptheta}}$ are:

8 $$- 2\frac{{\partial^{2} \ell \left( {{\varvec{\uptheta}}} \right)}}{{\partial {\uptheta }_{ij} \partial {\uptheta }_{kl} }} = - {\text{tr}}\left( {\frac{{\partial {\mathbf{V}}}}{{\partial {\uptheta }_{ij} }}{\mathbf{P}}\frac{{\partial {\mathbf{V}}}}{{\partial {\uptheta }_{kl} }}{\mathbf{P}}} \right) + 2 {\mathbf{y^{\prime}P}}\frac{{\partial {\mathbf{V}}}}{{\partial {\uptheta }_{ij} }}{\mathbf{P}}\frac{{\partial {\mathbf{V}}}}{{\partial {\uptheta }_{kl} }}{\mathbf{Py}}.$$

In practice, neither Eq. (8) nor its expected value are used in practice due to computational reasons. In fact, they are replaced by the Average Information (AI) matrix [14]:

9 $${\mathbf{AI}}\left( {{\uptheta }_{ij} ,{\uptheta }_{kl} } \right) = {\mathbf{y^{\prime}P}}\frac{{\partial {\mathbf{V}}}}{{\partial {\uptheta }_{ij} }}{\mathbf{P}}\frac{{\partial {\mathbf{V}}}}{{\partial {\uptheta }_{kl} }}{\mathbf{Py}}.$$

For EM REML, Eqs. (4) and (7) are set to zero and solved for the parameters of interest [15]. For AI REML, the parameters for the iteration $i+1$ are obtained as $\text{vech}\left({{\varvec{\uptheta}}}^{i+1}\right)=\text{vech}\left({{\varvec{\uptheta}}}^{i}\right)-{{\varvec{\Delta}}}^{i}$ [14], where ${{\varvec{\Delta}}}^{i}=\mathbf{A}\mathbf{I}{\left({{\varvec{\uptheta}}}^{i}\right)}^{-1}\left(\nabla \left({{\varvec{\uptheta}}}^{i}\right)\right)$, $\nabla \left({{\varvec{\uptheta}}}^{i}\right)$ is the gradient of $-2{\ell}\left({\varvec{\uptheta}}\right)$ evaluated at ${{\varvec{\uptheta}}}^{i}$ and obtained from Eq. (4). Note that since ${\varvec{\uptheta}}$ is symmetric, only the elements of its upper or lower triangle are used in ${{\varvec{\Delta}}}^{i}$ for the iteration step.

For both EM and AI REML, the most time-consuming step is inverting $\mathbf{C}$, which is needed for ${\mathbf{T}}_{k}$ in Eq. (4) and $\text{tr}\left({\mathbf{R}}_{l}^{-1}{\mathbf{R}}_{ij}{\mathbf{R}}_{l}^{-1}\mathbf{Q}{\mathbf{C}}^{-1}{\mathbf{Q}}^{\prime}\right)$ in Eq. (7). In MC-ss-GREML, these are obtained using Monte Carlo simulation as detailed next.

### Trace calculation

The traces in Eqs. (4) and (7) could be obtained by a Monte Carlo or Hutchinson estimator [16], as outlined in [4] and [5]. The estimator relies on the expectation of a quadratic form: $\text{tr}\left(\mathbf{T}\mathbf{C}\right)=\text{E}\left({\mathbf{c}}^{\mathbf{{\prime}}}\mathbf{T}\mathbf{c}\right)$ for $\text{E}\left(\mathbf{c}\right)=0$ and $\text{Var}\left(\mathbf{c}\right)=\mathbf{C}$. As Hutchinson [16] showed, this can be estimated unbiasedly by a Monte Carlo estimator ${n}^{-1}{\sum }_{i=1}^{n}{\widetilde{\mathbf{c}}}_{i}^{\prime}\mathbf{T}{\widetilde{\mathbf{c}}}_{i}$, where $n$ vectors ${\widetilde{\mathbf{c}}}_{i}$ are drawn such that $\text{Var}\left({\widetilde{\mathbf{c}}}_{i}\right)=\mathbf{C}$. For that, random effects $\left(\widetilde{\mathbf{k}}\right)$, errors $\left(\widetilde{\mathbf{e}}\right)$, and phenotypes $\left(\widetilde{\mathbf{y}}\right)$ must be simulated, and the model in Eq. (1) must be solved with the simulated values. The simulated quantities are drawn as: $\widetilde{\mathbf{k}}\sim N\left(0,{{\varvec{\uptheta}}}_{k}\otimes \mathbf{K}\right)$, $\widetilde{\mathbf{e}}\sim N\left(0,{\mathbf{R}}_{l}\otimes \mathbf{I}\right)$, and $\widetilde{\mathbf{y}}=\sum {\widetilde{\mathbf{k}}}_{i}+\widetilde{\mathbf{e}}$, whereas solving generates simulated estimates and residuals, e.g. $\widehat{\widetilde{\mathbf{k}}}$ or $\widehat{\widetilde{\mathbf{e}}}$, that follow their expected sampling distributions.

For random effects except the error, ${\mathbf{T}}_{{k}_{ij}}$ is therefore approximated as:

10 $${\mathbf{T}}_{{k_{ij} }} \approx {\hat{\mathbf{T}}} _{{k_{ij} }}^{1} = q_{k}\uptheta _{{k_{ij} }} - s^{ - 1} \mathop \sum \limits_{l = 1}^{s} \widehat{{{\tilde{\mathbf{k}}}}}_{{i_{l} }}^{\prime} {\mathbf{K}}^{ - 1} \widehat{{{\tilde{\mathbf{k}}}}}_{{j_{l} }}$$

or:

11 $${\mathbf{T}}_{{k_{ij} }} \approx {\hat{\mathbf{T}}} _{{k_{ij} }}^{2} = s^{ - 1} \mathop \sum \limits_{l = 1}^{s} \left( {{\tilde{\mathbf{k}}}_{{i_{l} }} - \widehat{{{\tilde{\mathbf{k}}}}}_{{i_{l} }} } \right)^{\prime} {\mathbf{K}}^{ - 1} \left( {{\tilde{\mathbf{k}}}_{{j_{l} }} - \widehat{{{\tilde{\mathbf{k}}}}}_{{j_{l} }} } \right).$$

On the other hand, $\text{tr}\left({\mathbf{R}}_{l}^{-1}{\mathbf{R}}_{ij}{\mathbf{R}}_{l}^{-1}\mathbf{Q}{\mathbf{C}}^{-1}{\mathbf{Q}}{\prime}\right)$ is approximated as:

12 $${\text{tr}}\left( {{\mathbf{R}}_{l}^{ - 1} {\mathbf{R}}_{ij} {\mathbf{R}}_{l}^{ - 1} {\mathbf{QC}}^{ - 1} {\mathbf{Q^{\prime}}}} \right) \approx \; \; \; n_{ij} {\mathbf{R}}_{{l_{ij} }} - s^{ - 1} \mathop \sum \limits_{m = 1}^{s} \widehat{{{\tilde{\mathbf{e}}}}}_{{i_{m} }}^{\prime} {\mathbf{K}}^{ - 1} \widehat{{{\tilde{\mathbf{e}}}}}_{{j_{m} }} ,$$

or:

13 $${\text{tr}}\left( {{\mathbf{R}}_{l}^{ - 1} {\mathbf{R}}_{ij} {\mathbf{R}}_{l}^{ - 1} {\mathbf{QC}}^{ - 1} {\mathbf{Q^{\prime}}}} \right) \approx  \; \; \; s^{ - 1} \mathop \sum \limits_{m = 1}^{s} \left( {{\tilde{\mathbf{e}}}_{{i_{m} }} - \left( {{\tilde{\mathbf{y}}}_{{i_{m} }} - {\hat{\mathbf{y}}}_{{i_{m} }} } \right)} \right)^{\prime} \left( {{\tilde{\mathbf{e}}}_{{j_{m} }} - \left( {{\tilde{\mathbf{y}}}_{{j_{m} }} - {\hat{\mathbf{y}}}_{{j_{m} }} } \right)} \right).$$

For ssGBLUP, calculating quadratic forms and traces requires a product of the type ${\mathbf{x^{\prime}}}{\mathbf{H}}^{-1}\mathbf{x}$, where $\mathbf{x}$ is a vector. Since ${\mathbf{H}}^{-1}$ may not be explicitly available, ${\mathbf{x^{\prime}}}{\mathbf{H}}^{-1}\mathbf{x}$ should be calculated as ${\mathbf{x^{\prime}}}{\mathbf{A}}^{-1}\mathbf{x}+{\mathbf{x}}_{2}^{\mathbf{{\prime}}}{\mathbf{G}}^{-1}{\mathbf{x}}_{2}-{\mathbf{x}}_{2}^{\mathbf{{\prime}}}{\mathbf{A}}_{22}^{-1}{\mathbf{x}}_{2}$, where ${\mathbf{x}}_{2}$ is the block of $\mathbf{x}$ pertaining to the genotyped animals. The product ${\mathbf{x}}^{\mathbf{{\prime}}}{\mathbf{A}}^{-1}\mathbf{x}$ can be easily calculated by storing ${\mathbf{A}}^{-1}$ as a sparse matrix, or by Henderson’s rules [17]. The product ${\mathbf{x}}_{2}^{\mathbf{{\prime}}}{\mathbf{G}}^{-1}{\mathbf{x}}_{2}$ can be obtained explicitly if ${\mathbf{G}}^{-1}$ is available or by solving the linear system $\mathbf{G}\mathbf{w}={\mathbf{x}}_{2}$, using the Woodbury Identity [18], or approximated with the Algorithm for Proven and Young (APY; [19]), which uses a sparse representation of ${\mathbf{G}}^{-1}$. For genomic BLUP (GBLUP) models, the strategies outlined in the previous sentence could be applied. Finally, the product ${\mathbf{x}}_{2}^{\mathbf{{\prime}}}{\mathbf{A}}_{22}^{-1}{\mathbf{x}}_{2}$ can be calculated using sparse computations as in Masuda et al. [20]. It is worth noticing that an expanded version of ${\mathbf{H}}^{-1}$ could be used to avoid calculating ${\mathbf{G}}^{-1}$ or its variants [21].

### Simulation of breeding values

Let ${\mathbf{G}}_{0}$ denote the genetic (co)variance matrix at a specific iteration round, and $n$ be the number of genotyped animals. In most single-step models, $\mathbf{u}$ is defined as:

14 $$\left[ {\begin{array}{*{20}c} {{\mathbf{u}}_{1} } \\ {{\mathbf{u}}_{2} } \\ \end{array} } \right] = \sqrt {1 - \alpha } \left( {\left[ {\begin{array}{*{20}c} {{\mathbf{A}}_{12} {\mathbf{A}}_{22}^{ - 1} {\mathbf{T}}} \\ {\mathbf{T}} \\ \end{array} } \right]{\varvec{\mu}} + \left[ {\begin{array}{*{20}c} {{\mathbf{A}}_{12} {\mathbf{A}}_{22}^{ - 1} {\mathbf{M}}} \\ {\mathbf{M}} \\ \end{array} } \right]{\mathbf{g}}} \right) + \left[ {\begin{array}{*{20}c} {{\varvec{\upxi}}} \\ {\mathbf{0}} \\ \end{array} } \right] + \sqrt \alpha \left[ {\begin{array}{*{20}c} {{\mathbf{A}}_{12} {\mathbf{A}}_{22}^{ - 1} } \\ {\mathbf{I}} \\ \end{array} } \right]{{\varvec{\upvarepsilon}}},$$

where the subscript (1)2 refers to the (non)genotyped animals, $\alpha$ is the proportion of residual polygenic effects, ${\varvec{\mu}}$ is the vector of mean genetic value of ${\mathbf{u}}_{2}$ on the scale of the whole population [22] distributed as ${\varvec{\mu}}\sim N\left(0,{\mathbf{G}}_{0}{n}^{-2}{\mathbf{1}}^{\boldsymbol{{\prime}}}\left({\mathbf{A}}_{22}-\mathbf{G}\right)\mathbf{1}\right)$ whose incidence matrix is $\mathbf{T}$, $\mathbf{A}$ is the numerator relationship matrix, $\mathbf{M}$ is the matrix of centered genotypes, $\mathbf{g}$ is the vector of marker effects distributed as $\mathbf{g}\sim N\left(0,{\mathbf{G}}_{0}\otimes {\left(2{\sum }_{i}{p}_{i}{q}_{i}\right)}^{-1}\mathbf{I}\right)$, where ${p}_{i}$ is the allele frequency of the reference allele for the *i*^*th*^ locus and ${q}_{i}=1-{p}_{i}$, ${\varvec{\upxi}}$ is the vector of imputation errors [23] distributed as ${\varvec{\upxi}}\sim N\left(0,{\mathbf{G}}_{0}\otimes {\left({\mathbf{A}}^{11}\right)}^{-1}\right)$, and ${\varvec{\upvarepsilon}}$ is the vector of random polygenic effects distributed as ${\varvec{\upvarepsilon}}\sim N\left(0,{\mathbf{G}}_{0}\otimes {\mathbf{A}}_{22}\right)$. Other assumptions are possible, for instance, using metafounders [24] would expand $\mathbf{A}$ in (14) and drop the term ${\varvec{\mu}}$, whereas the use of multiple J-factors [25] would contain a vector of them in (14) instead of a single $\mu$ per trait. Also, a different variance for the marker effects can be assumed. Given the formulation in Eq. (14), the genomic relationship matrix is $\mathbf{G}=\sqrt{1-\alpha }\left(\mathbf{1}{\mathbf{1}}^{\boldsymbol{{\prime}}}{n}^{-2}{1}^{\boldsymbol{{\prime}}}\left({\mathbf{A}}_{22}-\mathbf{G}\right)\mathbf{1}+{\left(2\sum pq\right)}^{-1}\mathbf{M}{\mathbf{M^{\prime}}}\right)+\sqrt{\alpha }\boldsymbol{ }{\mathbf{A}}_{22}$. For different genomic relationship matrices (e.g., heterogeneous variances for marker effects, multiple J-factors, etc.), Eq. (14) can be modified accordingly.

Following the simulation scheme proposed by Pimentel et al. [8], Eq. (14) turns into:

15 $$\left[ {\begin{array}{*{20}c} {{\mathbf{u}}_{1} } \\ {{\mathbf{u}}_{2} } \\ \end{array} } \right] = \sqrt {1 - \alpha } \left( {\left[ {\begin{array}{*{20}c} {{\mathbf{A}}_{12} {\mathbf{A}}_{22}^{ - 1} {\mathbf{1}}} \\ {\mathbf{1}} \\ \end{array} } \right]\mu + \left[ {\begin{array}{*{20}c} {{\mathbf{A}}_{12} {\mathbf{A}}_{22}^{ - 1} {\mathbf{M}}} \\ {\mathbf{M}} \\ \end{array} } \right]{\mathbf{g}}} \right) + \left[ {\begin{array}{*{20}c} {{\mathbf{a}}_{1} - {\mathbf{A}}_{12} {\mathbf{A}}_{22}^{ - 1} {\mathbf{a}}_{2} } \\ {\mathbf{0}} \\ \end{array} } \right] + \sqrt \alpha \left[ {\begin{array}{*{20}c} {{\mathbf{A}}_{12} {\mathbf{A}}_{22}^{ - 1} } \\ {\mathbf{I}} \\ \end{array} } \right]{\mathbf{a}}_{2} ,$$

where $\mathbf{a}$ denotes breeding values simulated without genomic information. Rearranging terms:

16 $$\left[ {\begin{array}{*{20}c} {{\mathbf{u}}_{1} } \\ {{\mathbf{u}}_{2} } \\ \end{array} } \right] = \left[ {\begin{array}{*{20}c} {{\mathbf{A}}_{12} {\mathbf{A}}_{22}^{ - 1} \left( {\sqrt {1 - \alpha } \left( {{\mathbf{T}}{\varvec{\mu}} + {\mathbf{Mg}}} \right) + {\mathbf{a}}_{2} \left( {\sqrt \alpha - 1} \right)} \right) + {\mathbf{a}}_{1} } \\ {\sqrt {1 - \alpha } \left( {{\mathbf{T}}{\varvec{\mu}} + {\mathbf{Mg}}} \right) + \sqrt \alpha \user2{ }{\mathbf{a}}_{2} } \\ \end{array} } \right].$$

Let $\widetilde{\mathbf{x}}$ be a vector sampled from $N\left(0,\mathbf{I}\right)$ of size number of traits times number of correlated random effects. Note that the values of $\widetilde{\mathbf{x}}$ change each time it is reused. Then, based on Eq. (16), the algorithm for sampling $\widetilde{\mathbf{u}}\sim N\left(0,{\mathbf{G}}_{0}\otimes \mathbf{H}\right)$ is:

1. For the *i*th animal, simulate breeding values without genomic information as ${\mathbf{a}}_{i}=0.5\left({\mathbf{a}}_{{s}_{i}}+{\mathbf{a}}_{{d}_{i}}\right)+\mathbf{L}\widetilde{\mathbf{x}} \sqrt{{\phi }_{i}}$, where $0.5\left({\mathbf{a}}_{{s}_{i}}+{\mathbf{a}}_{{d}_{i}}\right)$ is the parent average, $\mathbf{L}{\mathbf{L^{\prime}}}={\mathbf{G}}_{0}$, and ${\phi }_{i}$ is the variance of the Mendelian sampling term for that individual.
2. For the *i*th locus, sample the *i*th group of marker effects as ${\mathbf{g}}_{i}=\mathbf{L}\widetilde{\mathbf{x}}{\left(2{\sum }_{i}{p}_{i}{q}_{i}\right)}^{-\frac{1}{2}}$. Here, different covariance structures can be used for marker effects.
3. Sample $${\varvec{\mu}}=\mathbf{L}\widetilde{\mathbf{x}}\sqrt{{n}^{-2}{\mathbf{1}}^{\boldsymbol{{\prime}}}\left({\mathbf{A}}_{22}-\mathbf{G}\right)\mathbf{1}}$$.
4. For the genotyped animals, calculate ${\widetilde{\mathbf{u}}}_{2}=\sqrt{1-\alpha }\left(\mathbf{T}{\varvec{\mu}}+\mathbf{M}\mathbf{g}\right)+\sqrt{\alpha }\boldsymbol{ }{\mathbf{a}}_{2}$ and ${\varvec{\updelta}}=\sqrt{1-\alpha }\left(\mathbf{T}{\varvec{\mu}}+\mathbf{M}\mathbf{g}\right)+\left(\sqrt{\alpha }-1\right){\mathbf{a}}_{2}$.
5. For the non-genotyped animals, calculate ${\widetilde{\mathbf{u}}}_{1}={\mathbf{A}}_{12}{\mathbf{A}}_{22}^{-1}{\varvec{\updelta}}+{\mathbf{a}}_{1}$.

Note that for GBLUP models, Eqs. (14–16) reduce to only genotyped animals and the algorithm for sampling $\widetilde{\mathbf{u}}$ just involves steps 2 and 4.

This algorithm does not involve matrix $\mathbf{G}$ or its inverse. However, using APY for the later structure of ${\mathbf{H}}^{-1}$ and solving the MME will reduce computing times while obtaining estimates of variance components that are very close to those of ssGBLUP without APY. In that case, it is necessary to consider the proper variance structure of core $\left(\text{c}\right)$ and non-core $\left(\text{n}\right)$ animals in APY. Following formulas 1–3 of Bermann et al. [26], Eq. (14) turns into:

17 $$\left[ {\begin{array}{*{20}c} {{\mathbf{u}}_{1} } \\ {{\mathbf{u}}_{{\text{n}}} } \\ {{\mathbf{u}}_{{\text{c}}} } \\ \end{array} } \right] = \sqrt {1 - \alpha } \left( {\left[ {\begin{array}{*{20}c} {{\mathbf{A}}_{12} {\mathbf{A}}_{22}^{ - 1} {\mathbf{1}}^{\user2{*}} } \\ {{\mathbf{P}}_{{{\text{nc}}}} {\mathbf{T}}} \\ {\mathbf{T}} \\ \end{array} } \right]{\varvec{\mu}} + \left[ {\begin{array}{*{20}c} {{\mathbf{A}}_{12} {\mathbf{A}}_{22}^{ - 1} {\mathbf{M}}^{*} } \\ {{\mathbf{P}}_{{{\text{nc}}}} {\mathbf{M}}_{{\text{c}}} } \\ {{\mathbf{M}}_{{\text{c}}} } \\ \end{array} } \right]{\mathbf{g}}} \right) + \left[ {\begin{array}{*{20}c} {{\varvec{\upxi}}} \\ {\mathbf{0}} \\ {\mathbf{0}} \\ \end{array} } \right] + \sqrt \alpha \left[ {\begin{array}{*{20}c} {{\mathbf{A}}_{12} {\mathbf{A}}_{22}^{ - 1} {\mathbf{P}}^{*} } \\ {{\mathbf{P}}_{{{\text{nc}}}} } \\ {\mathbf{I}} \\ \end{array} } \right]{{\varvec{\upvarepsilon}}}_{{\text{c}}} + \left[ {\begin{array}{*{20}c} {\mathbf{0}} \\ {{\varvec{\uppsi}}} \\ {\mathbf{0}} \\ \end{array} } \right],$$

where${\mathbf{P}}_{\text{nc}}={\mathbf{G}}_{\text{nc}}{\mathbf{G}}_{\text{cc}}^{-1}$,$${1}^{*}=\left[\begin{array}{c}{\mathbf{P}}_{\text{nc}}\mathbf{T}\\ \mathbf{T}\end{array}\right]$$,$${\mathbf{M}}^{*}=\left[\begin{array}{c}{\mathbf{P}}_{\text{nc}}{\mathbf{M}}_{\text{c}}\\ {\mathbf{M}}_{\text{c}}\end{array}\right]$$, $${\mathbf{P}}^{*}=\left[\begin{array}{c}{\mathbf{P}}_{\text{nc}}\\ 1\end{array}\right]$$, and ${\varvec{\uppsi}}\sim N\left(0,{\mathbf{G}}_{0}\otimes{\varvec{\Psi}}\right)$ is the error term of non-core animals, where ${\varvec{\Psi}}=diag\left({\mathbf{G}}_{\text{nn}}-{\mathbf{G}}_{\text{nc}}{\mathbf{G}}_{\text{cc}}^{-1}{\mathbf{G}}_{\text{cn}}\right)$ is a diagonal matrix and already includes a residual polygenic component inside each block of$\mathbf{G}$. By the same reasoning in Eq. (15):

18 $$\left[ {\begin{array}{*{20}c} {{\mathbf{u}}_{1} } \\ {{\mathbf{u}}_{{\text{n}}} } \\ {{\mathbf{u}}_{{\text{c}}} } \\ \end{array} } \right] = \sqrt {1 - \alpha } \left( {\left[ {\begin{array}{*{20}c} {{\mathbf{A}}_{12} {\mathbf{A}}_{22}^{ - 1} {\mathbf{1}}^{\user2{*}} } \\ {{\mathbf{P}}_{{{\text{nc}}}} {\mathbf{1}}} \\ {\mathbf{1}} \\ \end{array} } \right]\mu + \left[ {\begin{array}{*{20}c} {{\mathbf{A}}_{12} {\mathbf{A}}_{22}^{ - 1} {\mathbf{M}}^{*} } \\ {{\mathbf{P}}_{{{\text{nc}}}} {\mathbf{M}}_{{\text{c}}} } \\ {{\mathbf{M}}_{{\text{c}}} } \\ \end{array} } \right]{\mathbf{g}}} \right) + \left[ {\begin{array}{*{20}c} {{\mathbf{a}}_{1} - {\mathbf{A}}_{12} {\mathbf{A}}_{22}^{ - 1} {\mathbf{a}}_{2} } \\ {\mathbf{0}} \\ {\mathbf{0}} \\ \end{array} } \right] + \sqrt \alpha \left[ {\begin{array}{*{20}c} {{\mathbf{A}}_{12} {\mathbf{A}}_{22}^{ - 1} {\mathbf{P}}^{*} } \\ {{\mathbf{P}}_{{{\text{nc}}}} } \\ {\mathbf{I}} \\ \end{array} } \right]{\mathbf{a}}_{{\text{c}}} + \left[ {\begin{array}{*{20}c} {\mathbf{0}} \\ {{\varvec{\uppsi}}} \\ {\mathbf{0}} \\ \end{array} } \right].$$

Rearranging terms:

19 $$\left[ {\begin{array}{*{20}c} {{\mathbf{u}}_{1} } \\ {{\mathbf{u}}_{{\text{n}}} } \\ {{\mathbf{u}}_{{\text{c}}} } \\ \end{array} } \right] = \left[ {\begin{array}{*{20}c} {{\mathbf{A}}_{12} {\mathbf{A}}_{22}^{ - 1} \left( {\sqrt {1 - \alpha } \left( {{\mathbf{1}}^{\user2{*}} \mu + {\mathbf{M}}^{*} {\mathbf{g}}} \right) + \sqrt \alpha {\mathbf{P}}^{*} {\mathbf{a}}_{{\text{c}}} - {\mathbf{a}}_{2} } \right) + {\mathbf{a}}_{1} } \\ {{\mathbf{P}}_{{{\text{nc}}}} \left( {\sqrt {1 - \alpha } \left( {{\mathbf{1}}\mu + {\mathbf{M}}_{{\text{c}}} {\mathbf{g}}} \right) + \sqrt \alpha \user2{ }{\mathbf{a}}_{{\text{c}}} } \right) + {{\varvec{\uppsi}}}} \\ {\sqrt {1 - \alpha } \left( {{\mathbf{1}}\mu + {\mathbf{M}}_{{\text{c}}} {\mathbf{g}}} \right) + \sqrt \alpha \user2{ }{\mathbf{a}}_{{\text{c}}} } \\ \end{array} } \right].$$

Then, the 4th and 5th steps from the previous procedure are replaced as follows:

4. For the core animals, calculate ${\widetilde{\mathbf{u}}}_{\text{c}}=\sqrt{1-\alpha }\left(\mathbf{T}{\varvec{\mu}}+{\mathbf{M}}_{\text{c}}\mathbf{g}\right)+\sqrt{\alpha }\boldsymbol{ }{\mathbf{a}}_{\text{c}}$ and ${{\varvec{\updelta}}}_{\text{c}}=\sqrt{1-\alpha }\left(\mathbf{T}{\varvec{\mu}}+{\mathbf{M}}_{\text{c}}\mathbf{g}\right)+\left(\sqrt{\alpha }-1\right){\mathbf{a}}_{\text{c}}$.
5. For the non-core animals, sample ${{\varvec{\uppsi}}}_{i}=\mathbf{L}\widetilde{\mathbf{x}} \sqrt{{{\varvec{\Psi}}}_{ii}}$. Calculate ${\widetilde{\mathbf{u}}}_{\text{n}}={\mathbf{P}}_{\text{nc}}{\widetilde{\mathbf{u}}}_{\text{c}}$ and ${{\varvec{\updelta}}}_{\text{n}}={\widetilde{\mathbf{u}}}_{\text{n}}-{\mathbf{a}}_{\text{n}}$.
6. For the non-genotyped animals, calculate ${\widetilde{\mathbf{u}}}_{1}={\mathbf{A}}_{12}{\mathbf{A}}_{22}^{-1}{\varvec{\updelta}}+{\mathbf{a}}_{1}$.

### Convergence criterion

Both EM and AI REML proceed iteratively updating the estimates of the parameters until they reach convergence. The convergence of MC-REML cannot be assessed by the usual round-to-round relative difference $\left(\Delta {\varvec{\uptheta}}\right)$ because of the Monte Carlo error. Other approaches involve inspecting the changes of the log-likelihood from round to round [27]. Matilainen et al. [5] suggested combining the convergence criterion with the change in variance components predictions based on linear regression of the previous estimates $\left(C{C}_{E}^{k+1}\right)$. In a recent study, we presented a fast method to approximate the (restricted) log-likelihood function [28]. Therefore, in this study, we propose coupling the classical round-to-round relative difference with the coefficient of variation of the five previous rounds of the approximated restricted log-likelihood $\left(\text{CV}\left(\text{logL}\right)\right)$. In this setting, $\Delta {\varvec{\uptheta}}$ would keep a stringent stopping threshold (say, 10^–12^), whereas $\text{CV}\left(\text{logL}\right)$ would have a less strict convergence criterion (10^–4^). The log-likelihood approximation must always be performed with the same sequence of random numbers; thus, its variation will only occur due to change in variance components and not due to Monte Carlo error.

### Algorithm outline

The algorithm works using two thresholds. The first $\left({t}_{1}\right)$, is the usual one for the classical $\Delta {\varvec{\uptheta}}$, which is equal to 10^–12^. The second $\left({t}_{2}\right)$, is the stopping criterion for $\text{CV}\left(\text{logL}\right)$, which is 10^–4^.

The steps for one MC-REML iteration are:

1. Solve the MME using iteration on data [29–31].
2. Calculate quadratic forms in Eqs. (6) and (7).
3. For $s$ Monte Carlo samples, simulate random effects, residuals, and phenotypes and solve the MME to obtain simulated solutions.
4. Approximate traces based on Eqs. (10–13).
5. For AI-MC-REML, calculate the Average Information matrix as in Eq. (9) or solve an augmented MME as proposed by Thompson et al. [32] and Strandén et al. [33].
6. Obtain new estimates for variance components.
7. Approximate the log-likelihood using Bermann et al. [28] and calculate $\text{CV}\left(\text{logL}\right)$.
8. Finish the iteration if $\Delta {\varvec{\uptheta}}<{t}_{1}$ or $\text{CV}\left(\text{logL}\right)<{t}_{2}$.

## Materials

We tested the MC-ss-GREML with a three-trait model in beef cattle. The data were provided by the American Angus Association (St. Joseph, MO). Traits were birth weight (BW), weaning weight (WW), and post-weaning weight (PWG). The model was as follows:

20 $$\left[ {\begin{array}{*{20}c} {{\mathbf{y}}_{{{\text{bw}}}} } \\ {{\mathbf{y}}_{{{\text{ww}}}} } \\ {{\mathbf{y}}_{{{\text{pwg}}}} } \\ \end{array} } \right] = {\mathbf{Xb}} + \left[ {\begin{array}{*{20}c} {{\mathbf{Z}}_{{{\text{bw}}}} } & {\mathbf{0}} & {\mathbf{0}} \\ {\mathbf{0}} & {{\mathbf{Z}}_{{{\text{ww}}}} } & {\mathbf{0}} \\ {\mathbf{0}} & {\mathbf{0}} & {{\mathbf{Z}}_{{{\text{pwg}}}} } \\ \end{array} } \right]\left[ {\begin{array}{*{20}c} {{\mathbf{u}}_{{{\text{bw}}}} } \\ {{\mathbf{u}}_{{{\text{ww}}}} } \\ {{\mathbf{u}}_{{{\text{pwg}}}} } \\ \end{array} } \right] + \left[ {\begin{array}{*{20}c} {{\mathbf{W}}_{{{\text{bw}}}} } & {\mathbf{0}} \\ {\mathbf{0}} & {{\mathbf{W}}_{{{\text{ww}}}} } \\ {\mathbf{0}} & {\mathbf{0}} \\ \end{array} } \right]\left[ {\begin{array}{*{20}c} {{\mathbf{m}}_{{{\text{bw}}}} } \\ {{\mathbf{m}}_{{{\text{ww}}}} } \\ \end{array} } \right] + \left[ {\begin{array}{*{20}c} {\mathbf{0}} \\ {{\mathbf{W}}_{{{\text{ww}}}} } \\ {\mathbf{0}} \\ \end{array} } \right]{\mathbf{mpe}}_{{{\mathbf{ww}}}} + {\mathbf{e}}$$

where $\mathbf{b}$ denotes the contemporary group effects, $\mathbf{u}$ the additive genetic effects, $\mathbf{m}$ the maternal genetic effects, $\mathbf{m}\mathbf{p}\mathbf{e}$ the maternal permanent environmental effect, and $\mathbf{e}$ the error. $\mathbf{X}$, $\mathbf{Z}$, and $\mathbf{W}$ are incidence matrices. All random effects are assumed to follow a multivariate normal distribution with null expectation. For the direct and maternal genetic effects, the following covariance structure is assumed:

21 $${\text{Var}}\left[ {\begin{array}{*{20}c} {{\mathbf{u}}_{{{\text{bw}}}} } \\ {{\mathbf{u}}_{{{\text{ww}}}} } \\ {{\mathbf{u}}_{{{\text{pwg}}}} } \\ {{\mathbf{m}}_{{{\text{bw}}}} } \\ {{\mathbf{m}}_{{{\text{ww}}}} } \\ \end{array} } \right] = \left[ {\begin{array}{*{20}c} {{\mathbf{K}}\sigma_{{{\text{u}}_{{{\text{bw}}}} }}^{2} } & {{\mathbf{K}}\sigma_{{{\text{u}}_{{{\text{bw}},{\text{ww}}}} }} } & {{\mathbf{K}}\sigma_{{{\text{u}}_{{{\text{bw}},{\text{pwg}}}} }} } & {{\mathbf{K}}\sigma_{{{\text{u}},{\text{m}}_{{{\text{bw}}}} }} } & {\mathbf{0}} \\ {} & {{\mathbf{K}}\sigma_{{{\text{u}}_{{{\text{ww}}}} }}^{2} } & {{\mathbf{K}}\sigma_{{{\text{u}}_{{{\text{ww}},{\text{pwg}}}} }} } & {\mathbf{0}} & {\mathbf{0}} \\ {} & {} & {{\mathbf{K}}\sigma_{{{\text{u}}_{{{\text{pwg}}}} }}^{2} } & {\mathbf{0}} & {\mathbf{0}} \\ {} & {symm} & {} & {{\mathbf{K}}\sigma_{{{\text{m}}_{{{\text{bw}}}} }}^{2} } & {\mathbf{0}} \\ {} & {} & {} & {} & {{\mathbf{K}}\sigma_{{{\text{m}}_{{{\text{ww}}}} }}^{2} } \\ \end{array} } \right]$$

where $\mathbf{K}$ could be either $\mathbf{A}$ for REML and MC-REML or $\mathbf{H}$ ss-GREML and MC-ss-GREML. The genomic relationship matrix was constructed as in the first method of [34] using observed allele frequencies and included a five percent of residual polygenic effect and one implicit J-factor as in Vitezica et al. [22]. The maternal permanent environmental effect is assumed to be uncorrelated with the other random effects with $\text{Var}\left(\mathbf{m}\mathbf{p}{\mathbf{e}}_{\mathbf{w}\mathbf{w}}\right)={\sigma }_{{\text{mpe}}_{\text{ww}}}^{2}$. The covariance structure for the error is $\text{Var}\left(\mathbf{e}\right)={\mathbf{R}}_{0}\otimes \mathbf{I}$, where:

22 $${\mathbf{R}}_{0} = \left[ {\begin{array}{*{20}c} {\sigma_{{{\text{e}}_{{{\text{bw}}}} }}^{2} } & {\sigma_{{{\text{e}}_{{{\text{bw}},{\text{ww}}}} }} } & 0 \\ {} & {\sigma_{{{\text{e}}_{{{\text{ww}}}} }}^{2} } & 0 \\ {symm} & {} & {\sigma_{{{\text{e}}_{{{\text{pwg}}}} }}^{2} } \\ \end{array} } \right]$$

Thus, the parameters to estimate are $\text{vech}\left({\varvec{\theta}}\right)={\left[{\sigma }_{{\text{u}}_{\text{bw}}}^{2} {\sigma }_{{\text{u}}_{\text{bw},\text{ww}}} {\sigma }_{{\text{u}}_{\text{ww}}}^{2} {\sigma }_{{\text{u}}_{\text{bw},\text{pwg}}} {\sigma }_{{\text{u}}_{\text{ww},\text{pwg}}} {\sigma }_{{\text{u}}_{\text{pwg}}}^{2} {\sigma }_{{\text{u},\text{m}}_{\text{bw}}} {\sigma }_{{\text{m}}_{\text{bw}}}^{2} {\sigma }_{{\text{m}}_{\text{ww}}}^{2} {\sigma }_{{\text{mpe}}_{\text{ww}}}^{2} {\sigma }_{{\text{e}}_{\text{bw}}}^{2} {\sigma }_{{\text{e}}_{\text{bw},\text{ww}}} {\sigma }_{{\text{e}}_{\text{ww}}}^{2} {\sigma }_{{\text{e}}_{\text{pwg}}}^{2}\right]}{\prime}$, giving a total of 14 parameters to estimate.

The residual covariance between PWG and the other traits is assumed to be zero by the American Angus Association to improve the convergence of their models due to the imbalance in the trait recording from weaning to yearling weight (André Garcia, American Angus Association, personal communication).

Two datasets were used. A small dataset was used to fit the model in Eq. (20), aiming to test the accuracy of MC-REML and MC-ss-GREML. The small dataset had 100,277 animals in the pedigree, from which 10,000 were genotyped. The number of records were 33,779, 33,595, and 33,595 for BW, WW, and PWG, respectively. The number of equations for the small dataset was 911,961. A large dataset was used to assess the computational performance of MC-REML and MC-ss-GREML. For the sake of time, we only fit the model for BW. Such dataset had 7,402,820 animals in the pedigree, from which 331,663 were genotyped and 5,788,373 had a record. APY was used, and the number of randomly selected core animals was equal to 15,000.

The number of Monte Carlo samples was empirically chosen such that when increasing it by five, no significant changes in estimates were observed. This resulted in 30 and 5 samples for the small and large datasets, respectively. Starting values were equal to one for all variances and 0.1 for covariances. We used Eq. (10) and (12) to approximate the traces because Eqs. (11) and (13) require more samples to achieve an accurate estimation [35]. The AI algorithm was used in all analyses without any starting rounds of EM. The computations for the exact traces used as a benchmark with the small dataset were done with the YAMS package [36]. All the analyses were carried out on a Dell PowerEdge R740XD server with 1.5 TB of memory, 45 TB of disk, and two Intel Xeon Gold 6258R processors, using four threads for the calculations.

## Results and discussion

The parameter estimates and respective standard errors for the small dataset are given in Table 1. We tested for significant differences between methods using a Wald test based on the inverse of the AI matrix and did not observe significant differences in estimates between any of the methods. Also, the point estimates from both methods were numerically close, except for the maternal genetic covariance between BW and WW $\left({\sigma }_{{\text{u},\text{m}}_{\text{bw}}}\right)$ when comparing ss-GREML and MC-ss-GREML. However, the overestimation by MC-ss-GREML was not statistically significant. We do acknowledge that the values shown in Table 1 might differ from those published by the American Angus Association (https://www.angus.org/tools-resources/national-cattle-evaluation/heritabilities-and-genetic-correlations), showing an extreme value for the additive genetic covariance between BW and PWG. Since this study aims to approximate REML variance components estimation in single-step models, the difference between those obtained with the exact and approximated methods is more important than point estimates. The difference between our estimates and the published ones is likely due to an artifact of data subsetting to estimate results in Table 1. Estimation of variance components with the large dataset is provided in Table 2. Although from a reduced model, these variance components were similar from those publicly available. Also, the reduction in the estimates’ standard errors with larger data can be observed when comparing Tables 1 and 2. For instance, the standard error of the residual variance for BW was reduced by almost ten times.

**Table 1:** Estimates of the variance components for the small dataset

Parameter	REML^1^	MC-REML^1^	ss-GREML	MC-ss-GREML
${\sigma }_{{\text{u}}_{\text{bw}}}^{2}$	25.71 (1.43)	25.77 (1.43)	24.90 (1.36)	23.98 (1.30)
${\sigma }_{{\text{u}}_{\text{bw},\text{ww}}}$	39.63 (4.45)	41.24 (4.47)	38.29 (4.32)	38.74 (4.30)
${\sigma }_{{\text{u}}_{\text{ww}}}^{2}$	368.78 (34.35)	391.45 (34.49)	380.19 (32.92)	378.44 (32.78)
${\sigma }_{{\text{u}}_{\text{bw},\text{pwg}}}$	52.18 (2.51)	51.85 (2.52)	52.51 (2.54)	52.04 (2.54)
${\sigma }_{{\text{u}}_{\text{ww},\text{pwg}}}$	49.32 (14.84)	48.50 (14.87)	34.94 (14.84)	36.01 (14.91)
${\sigma }_{{\text{u}}_{\text{pwg}}}^{2}$	577.72 (33.62)	581.71 (33.77)	560.17 (32.46)	575.40 (32.99)
${\sigma }_{{\text{u},\text{m}}_{\text{bw}}}$	− 0.99 (0.62)	− 1.00 (0.62)	− 0.82 (0.63)	− 0.46 (0.55)
${\sigma }_{{\text{m}}_{\text{bw}}}^{2}$	3.99 (0.64)	3.88 (0.63)	4.20 (0.60)	3.67 (0.58)
${\sigma }_{{\text{m}}_{\text{ww}}}^{2}$	198.94 (24.32)	203.58 (24.62)	184.51 (22.50)	184.74 (22.50)
${\sigma }_{{\text{mpe}}_{\text{ww}}}^{2}$	250.19 (32.55)	249.61 (32.58)	262.26 (31.85)	258.87 (31.87)
${\sigma }_{{\text{e}}_{\text{bw}}}^{2}$	30.44 (0.85)	30.49 (0.85)	30.91 (0.81)	31.58 (0.78)
${\sigma }_{{\text{e}}_{\text{bw},\text{ww}}}$	56.24 (3.23)	55.12 (3.23)	57.58 (3.12)	57.47 (3.11)
${\sigma }_{{\text{e}}_{\text{ww}}}^{2}$	1437.80 (35.58)	1430.30 (35.53)	1443.60 (35.00)	1448.40 (35.02)
${\sigma }_{{\text{e}}_{\text{pwg}}}^{2}$	1610.70 (26.13)	1608.50 (26.19)	1627.00 (25.19)	1620.40 (25.39)

Standard errors within parenthesis

The parameters for each row are additive genetic variance of birth weight (BW), additive genetic covariance between birth weight and weaning weight (WW), additive genetic variance of WW, additive genetic covariance between BW and post weaning gain (PWG), additive genetic variance of PWG, covariance between additive and maternal genetic effects for BW, maternal genetic variance of BW, maternal genetic variance of WW, maternal permanent environmental variance of WW, residual variance of BW, residual covariance between BW and WW, and residual variance of WW, residual variance of PWG, respectively

^1^ REML and MC-REML refer to the model without genomic information

**Table 2:** Estimates of the variance components for the large dataset

Parameter	MC-REML^1^	MC-ss-GREML
${\sigma }_{{\text{u}}_{\text{bw}}}^{2}$	30.70 (0.17)	29.22 (0.15)
${\sigma }_{{\text{u},\text{m}}_{\text{bw}}}$	− 4.48 (0.092)	− 3.73 (0.083)
${\sigma }_{{\text{m}}_{\text{bw}}}^{2}$	10.84 (0.069)	10.17 (0.066)
${\sigma }_{{\text{e}}_{\text{bw}}}^{2}$	29.38 (0.088)	30.46 (0.076)

Standard errors within parenthesis

The parameters for each row are additive genetic variance of birth weight (BW), covariance between additive and maternal genetic effects for BW, maternal genetic variance of BW and residual variance of BW, respectively

^1^ REML refers to the model without genomic information

To ensure that solutions remain in the parameter space, the Blupf90 software performs a weighted average of the AI and EM information matrices, as suggested by [13]. The same procedure was implemented for MC-REML and MC-ss-GREML. However, it was not required for our analysis because the solutions from each round of AI-REML always remained in the parameter space.

The method’s computing performance metrics for both datasets are provided in Table 3. As expected, for models without genomic information that could be inverted, the exact REML and Monte-Carlo REML showed similar performance. In contrast, MC-ss-GREML converged in 14% of the time required for ss-GREML. There is a notable reduction in memory and time requirements when replacing ss-GREML with MC-ss-GREML, which is explained by the fact that the MME are not stored in memory and no Cholesky factor is calculated. Note that the Cholesky factor requires more memory than the MME due to fill-ins. The timing for variance components estimation with the large dataset was very acceptable. In our case, the increase in computing time when adding genomics is mainly due to the calculation of ${\mathbf{A}}_{22}^{-1}{\mathbf{x}}_{2}={\mathbf{A}}^{22}{\mathbf{x}}_{2}-{\mathbf{A}}^{20}{\left({\mathbf{A}}^{00}\right)}^{-1}{\mathbf{A}}^{02}{\mathbf{x}}_{2}$ as in [20], where the superscript zero means non-genotyped ancestors, progeny, and mates of genotyped animals. ${\mathbf{A}}^{00}$ is factorized as a preprocessing step, and then whenever ${\mathbf{A}}_{22}^{-1}{\mathbf{x}}_{2}$ is needed, the linear system ${\mathbf{A}}^{00}{\mathbf{x}}_{0}={\mathbf{y}}_{0}$ is solved by forward–backward substitution. In the large dataset, ${\mathbf{A}}^{00}$ is of dimension 562,248 and has 1,823,479 non-zero elements. Due to the pedigree structure of genotyped animals, the number of elements of the Cholesky factor of ${\mathbf{A}}^{00}$ is 612,354,728 (due to fill-ins), which is a large number that can slow down the forward–backward substitution.

**Table 3:** Computing performance of variance components estimation with the small and large datasets

	Small dataset				Large dataset	
	REML^1^	MC-REML^1^	ss-GREML	MC-ss-GREML	MC-REML^1^	MC-ss-GREML
Rounds to converge	21	19	21	19	11	11
Peak memory usage in GB	3.3	0.4	105.5	1.1	14.3	53.3
Samples per round	–	30	–	30	5	5
Total time in hours	6.1	3.6	154.3	22.1	9.9	121.4

The small dataset was fitted to a three-trait model with 14 parameters to estimate. The large dataset was fitted to a single-trait model with 4 parameters to estimate.^1^ REML and MC-REML refer to the model without genomic information

As shown by Matilainen et al. [7], Monte Carlo REML methods based on second derivatives are sensitive to the number of samples, the sequence of random numbers used for the samples, and the convergence of the solvers. Monte Carlo REML based on second derivatives naturally suffers from instability because of the propagation of small errors in the approximated gradient [6]. Re-using the same sequence of pseudo-random numbers for sampling in each round removes such instability at the expense of some small bias [7]. As Matilainen et al. [7] suggested, we reused the same sequence in each round. Although the iterations can be smooth with that technique, reaching the classical convergence criteria $\left(\Delta {\varvec{\uptheta}}\right)$ might still be difficult due to small errors in the gradient resulting from its approximation. We observed that close to convergence, although a strict value (1e−12) of $\Delta {\varvec{\uptheta}}$ was not reached, the estimates were very similar, and the value of the restricted log-likelihood was not changing across rounds. Consequently, we found that the oscillation of AI-MC-REML close to convergence can be removed by using the variation of the restricted log-likelihood across rounds.

The computing time of AI-MC-ss-GREML can be roughly estimated as ${n}_{r}t\left(s\times n{r}_{s}+n{r}_{ai}+n{r}_{mme}\right)$, where ${n}_{r}$ is the number of rounds that AI-REML takes until convergence, $t$ is the time required for one preconditioned conjugated gradient (PCG) iteration, and $n{r}_{s}$, $n{r}_{ai}$, and $n{r}_{mme}$ are the number of PCG iterations until convergence for each sample, for solving the augmented MME [33], and for solving the MME, respectively. Note that the last three values are variable across AI-REML rounds due to the change in variance components, making the previous formula an approximation. Nonetheless, finding the minimum optimal number of samples is critical to ensure proper variance components estimation while keeping a reasonable computing time. In our study, when defining the optimal number of samples for the analysis, we observed that such a number varies with the number of traits, number of correlated random effects (direct and maternal, or only direct), and the inclusion of genomic information. However, it does not seem to be affected by the population size. Further studies to find the optimal number of Monte Carlo samples would be required to ensure an efficient implementation of MC-ss-GREML. In this study, we tried relaxing the stopping criterion for PCG and starting from previous solutions when solving the MME for each sample. While the first worked and reduced computational time, the second did not (results not shown). For datasets with a large number of genotyped animals, using APY with fewer core animals compared to regular evaluations could help reduce computing costs.

AI-REML requires the calculation of the AI matrix, which under classical algorithms requires solving the MME ${n}_{par}$ times, where ${n}_{par}$ is the number of variance components to estimate. Stranden et al. [33] revisited a method from [32], which proposed to replace solving ${n}_{par}$ times the MME by solving an augmented form of the MME only once, without explicit computation of the AI matrix. These augmented MME give as a solution ${{\varvec{\Delta}}}^{i}$; therefore, solving the MME ${n}_{par}$ times is avoided. This approach should be standard for models with many parameters to estimate and when the time for solving the MME is considerable. To obtain the standard errors of the parameters estimates, one can explicitly calculate the AI matrix at the end of the estimation.

Whether estimating variance components with large data and genomic information is really needed in practice is a valid discussion point. For very large populations under strong selection, even a small population sample could be too large to use exact REML algorithms; thus, using Monte Carlo REML is justified in those cases. Also, data subsetting in such cases is difficult depending on the number of years, type of selection, or underlying statistical model. Regarding genomic information, it is not clear if it should be included when estimating variance components. Some authors argued that including it produced biased results [37, 38], whereas others showed that there is no difference in including it or not and suggested that including it might be more appropriate [39]. Hidalgo et al. [40] showed that not including genomic information can result in biased variance components if the population is undergoing genomic selection. It is generally admitted that genetic trends are underestimated in absence of genomic information [41], but it is unclear (yet) if this poses problems for variance component estimation. On the other hand, if subsetting is used, the effect of genomic selection in such subpopulation might be higher than in the whole population, indicating that genotypes must be included to account for such genomic selection. Legarra [42] showed that estimates with and without genomic information are directly comparable when pedigree and relationship matrices align correctly, and not doing so is a frequent source of apparent disagreement between genomic and pedigree estimates. In any case, it is known that parameters estimated without genomic information are the expected value of those estimated with genomic information [43]. For populations without strong selection, a large proportion of genotyped animals, or genotyped at random, MC-REML and MC-ss-GREML should give similar results. However, if those conditions are not met, including genomic information when estimating genetic parameters could be of major importance.

## Conclusions

We developed a method for estimating variance components with REML in ssGBLUP models named MC-ss-GREML. The method gave similar estimates compared to exact ss-GREML while using considerably shorter computing time and much less memory. Since it uses iteration on data, MC-ss-GREML can be applied to routine genetic evaluation datasets.

## Data Availability

The data cannot be shared since it belongs to the American Angus Association (ST. Joseph, MO). The software that encapsulates MC-ss-GREML is currently available only under research agreement with the Animal Breeding and Genetics group at UGA (http://nce.ads.uga.edu/).
